# MnTnBuOE-2-PyP protects normal colorectal fibroblasts from radiation damage and simultaneously enhances radio/chemotherapeutic killing of colorectal cancer cells

**DOI:** 10.18632/oncotarget.8923

**Published:** 2016-04-22

**Authors:** Elizabeth A. Kosmacek, Arpita Chatterjee, Qiang Tong, Chi Lin, Rebecca E. Oberley

**Affiliations:** ^1^ Department of Biochemistry and Molecular Biology, University of Nebraska Medical Center, Omaha, NE, 68198, USA; ^2^ Department of Radiation Oncology, University of Nebraska Medical Center, Omaha, NE, 68198, USA

**Keywords:** MnTnBuOE-2-PyP, radiation, fibroblast, colorectal cancer, oxidative stress

## Abstract

Manganese porphyrins have been shown to be potent radioprotectors in a variety of cancer models. However, the mechanism as to how these porphyrins protect normal tissues from radiation damage still remains largely unknown. In the current study, we determine the effects of the manganese porphyrin, MnTnBuOE-2-PyP, on primary colorectal fibroblasts exposed to irradiation. We found that 2 Gy of radiation enhances the fibroblasts' ability to contract a collagen matrix, increases cell size and promotes cellular senesence. Treating fibroblasts with MnTnBuOE-2-PyP significantly inhibited radiation-induced collagen contraction, preserved cell morphology and also inhibited cellular senescence. We further showed that MnTnBuOE-2-PyP enhanced the overall viability of the fibroblasts following exposure to radiation but did not protect colorectal cancer cell viability. Specifically, MnTnBuOE-2-PyP in combination with irradiation, caused a significant decrease in tumor clonogenicity. Since locally advanced rectal cancers are treated with chemoradiation therapy followed by surgery and non-metastatic anal cancers are treated with chemoradiation therapy, we also investigated the effects of MnTnBuOE-2-PyP in combination with radiation, 5-fluorouracil with and without Mitomycin C. We found that MnTnBuOE-2-PyP in combination with Mitomycin C or 5-fluorouracil further enhances those compounds' ability to suppress tumor cell growth. When MnTnBuOE-2-PyP was combined with the two chemotherapeutics and radiation, we observed the greatest reduction in tumor cell growth. Therefore, these studies indicate that MnTnBuOE-2-PyP could be used as a potent radioprotector for normal tissue, while at the same time enhancing radiation and chemotherapy treatment for rectal and anal cancers.

## INTRODUCTION

The majority of rectal and anal cancer patients will be treated with some form of radiation therapy (American Cancer Society). Standard therapy for stage II and III rectal cancers includes radiation in combination with chemotherapy (5-fluorouracil) followed by surgery; standard therapy for localized anal cancer is radiation therapy in combination with chemotherapy (5-fluorouracil and Mitomycin C, NCCN guidelines). Most patients will suffer from acute and/or chronic side-effects associated with radiation exposure, which leads to a reduced quality of life [1]. For example, acute complications from radiation include diarrhea, skin soreness and bladder irritation [1]. Long-term complications from radiation therapy directed to the colorectal region are bowel fibrosis, rectal wall damage, bowel incontinence, rectal and bladder bleeding and pelvic fracture [1]. Currently, there are no FDA approved treatments to protect normal pelvic tissues from radiation damage. Radioprotectors are not used during rectal cancer radiotherapy because it is feared that the tumors will also be protected from radiation killing. The addition of an effective radioprotector while undergoing radiation therapy to treat rectal and anal cancers could drastically improve patient quality of life.

Radiation exposure leads to persistent inflammation in normal tissues caused by reactive oxygen species (ROS), such as superoxide (O_2_^•−^), which continues for months or years following radiation exposure [2]. The enhanced levels of ROS promote the initial tissue damage, mainly through cell death and acute inflammation [3, 4]. ROS also drive the chronic damage associated with radiation, mainly chronic inflammation and fibrosis [3–5]. Thus, inhibiting radiation-induced ROS could result in significant radioprotection against normal tissue injury.

The fibroblast is the cell that aberrantly lays down excess collagen in response to radiation [6]. An irradiated fibroblast can become epigenetically modified after radiation and display a myofibroblast phenotype [7]. These myofibroblasts are activated fibroblasts, which have increased cell size, increased contractibility, enhanced secretion of pro-fibrotic signals, and upregulated production of collagen and fibronectin [6]. Myofibroblast differentiation appears to be triggered in a part through TGFB1, IL-13, and IL-4 cytokine release [6].

Radiation can also cause cells to become senescent [8]. It has recently been shown that senescent cells, although metabolically quiescent, produce pro-inflammatory cytokines and help to maintain an inflammatory state [8–10]. Senescent cells have been shown to be involved in aging and the progression of other diseases, such as cancer, diabetes, obesity, and neurodegeneration [8]. Although not proven, recent studies suggest that senescent cells promote and maintain inflammation after radiation exposure.

ROS has been implicated in driving both cellular senescence and the promotion of the activated fibroblast (myofibroblast phenotype) [9, 11]. Therefore, we postulate that scavenging ROS may block the activated fibroblast phenotype and inhibit senescence of irradiated normal cells. Thus, scavenging of ROS would result in less inflammation and less fibrosis after radiation exposure to normal tissues.

Manganese porphyrins are a series of drugs that are catalytic scavengers of O_2_^•−^ and have been shown to protect from radiation damage in a variety of models [12–18]. Specifically, MnTE-2-PyP has been shown to protect the rectum from radiation damage. MnTE-2-PyP inhibits both acute and chronic effects associated with radiation [12]. A newer manganese porphyrin, MnTnBuOE-2-PyP has been shown to be an effective radioprotector in the context of head and neck cancer and glioblastomas [18, 19].

Useful radioprotectors should selectively protect normal tissues while leaving the cancer cells vulnerable to radiation-induced cell death. Cancer cells have increased production of ROS, relative to normal cells, which leads to uncontrolled cell proliferation and cancer progression [20–26]. Recent work has indicated that the manganese porphyrins act as a pro-oxidant in cancer cells by taking advantage of higher hydrogen peroxide levels, which results in the alteration of cell signaling and promotes cancer cell death [27–29]. Specifically, in lymphoma it has been shown that Mn porphyrins use H_2_O_2_ and glutathione to oxidize cysteines of signaling proteins [29]. In contrast, normal cells readily use redundant enzymes to maintain H_2_O_2_ at physiological nM levels. [30, 31]. Given the different levels of H_2_O_2_ in normal vs cancer cells, the yield of reaction with Mn porphyrins and H_2_O_2_ is different in those tissues and, thus, leads to different outcomes in the two cell types [31, 32].

In the present study, we investigate the effects of MnTnBuOE-2-PyP as a radioprotector of primary mouse colorectal fibroblasts. We show that MnTnBuOE-2-PyP significantly inhibits radiation induced activation of fibroblasts as measured by the collagen contraction assay and cell size. MnTnBuOE-2-PyP also significantly inhibits radiation-induced senescence. MnTnBuOE-2-PyP protects from radiation-induced fibroblast cell death but at the same time enhances colorectal cancer cell killing alone and in combination with radiation. Further, we show that the addition of MnTnBuOE-2-PyP with the standard colorectal cancer therapy (radiation, Mitomycin C and 5-fluorouracil) results in significantly more cancer cell killing than treatment with standard therapies alone. Thus, MnTnBuOE-2-PyP protects normal colorectal fibroblasts from radiation induced damage, while further inhibiting colorectal cancer growth in the presence of radiation and chemotherapy agents.

## RESULTS

The goal of this study was to understand the mechanisms by which MnTnBuOE-2-PyP works as a radioprotector. To begin answering this question we developed an *in vitro* model using primary mouse colorectal fibroblasts isolated from adult C57BL/6 mice. By day 5 in culture, all the cells stained positive for fibroblast markers and none displayed markers of epithelial cells (data not shown). Cells were cultured for 2 weeks before experimentation, maximizing the cell number at low enough population doublings to ensure optimal survival and minimal senescence or phenotypic change from culturing. For each isolation, half of the fibroblasts were placed in MnTnBuOE-2-PyP two days after the primary cells were isolated and remained in MnTnBuOE-2-PyP throughout the course of the experiment.

Pharmacokinetic analysis has been performed on MnTnBuOE-2-PyP and after a single subcutaneous injection of drug into a mouse, MnTnBuOE-2-PyP reaches the colon tissues at a concentration of 0.1-0.5 μM (data not shown). Therefore, we used *in vitro* concentrations of MnTnBuOE-2-PyP that would adequately reflect concentrations that would be achievable *in vivo*.

**Figure 1:**
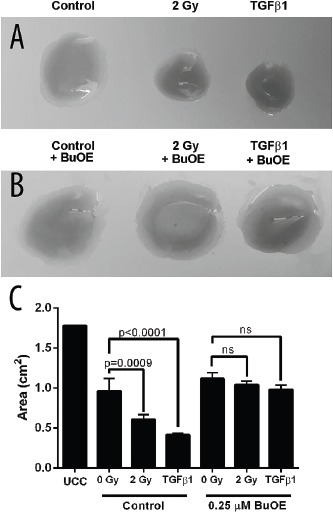
MnTnBuOE-2-PyP prevents contraction of collagen by primary colorectal fibroblasts Mouse primary colorectal fibroblasts were grown in rat tail collagen discs. **A.** Fibroblasts were treated with 2 Gy x-rays or 5 ng/mL TGFβ1. **B.** Fibroblasts grown in 0.25 μM MnTnBuOE-2-PyP (BuOE) and treated with 2 Gy x-rays or 5 ng/mL TGFβ1. **C.** Collagen disc area measurements. All data are representative of the mean and standard deviation and were obtained from 3 independent experiments. p-values and significance were determined using 1-way ANOVA followed by post hoc Tukey's test for multiple comparisons.

It has been shown in other models of fibrosis that when fibroblasts become activated, they are able to contract the matrix surrounding the cells. In previous models, the addition of TGF-β1 causes fibroblasts to contract *in vitro*. Radiation has also been shown to cause activation of fibroblasts. So, we treated colorectal fibroblasts then seeded them in rat tail collagen discs as a functional endpoint of activation following treatment with radiation or TGF-β1. We found that 48 hours after irradiating fibroblasts with 2 Gy or treatment with TGF-β1 (5 ng/mL) resulted in significant contraction of the rat tail collagen disc, indicating that these fibroblasts were activated (Figure 1A&1C). In contrast, the discs containing fibroblasts that were treated with MnTnBuOE-2-PyP and irradiated with 2 Gy of radiation or treated with TGF-β1 did not contract more than the non-treated control (Figure 1B&1C). Thus, MnTnBuOE-2-PyP treatment inhibited the activation of the fibroblasts by radiation and TGF-β1 stimulation.

**Figure 2:**
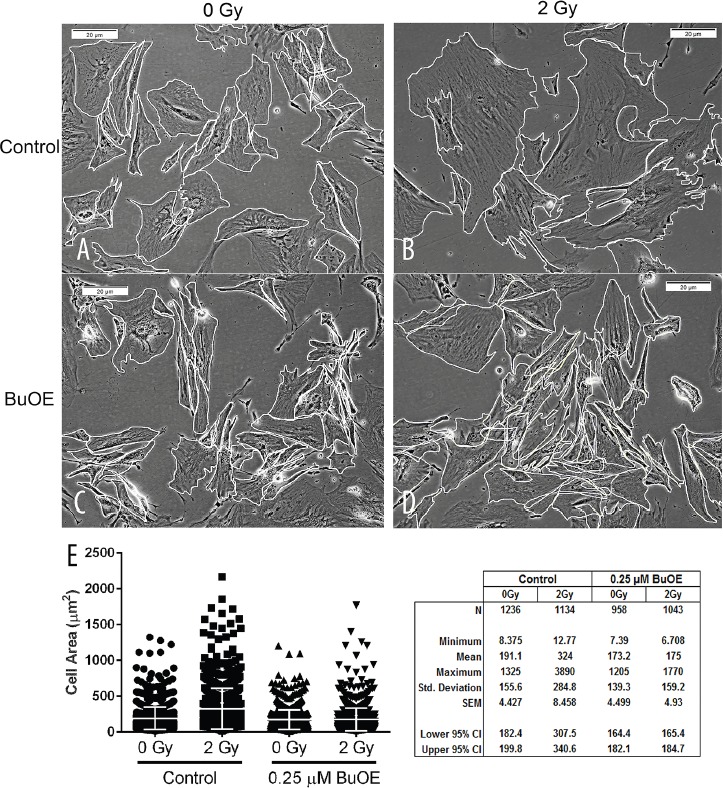
MnTnBuOE-2-PyP mitigates radiation-induced increase in cell size of primary colorectal fibroblasts Images of mouse colorectal fibroblasts maintained in media with PBS (Control) or 0.25 μM MnTnBuOE-2-PyP (BuOE) were either sham irradiated **A** & **C.** or treated with 2 Gy x-rays **B** & **D.** then harvested 48 hours later. After cells were imaged, their borders were manually traced in ImageJ to calculate cell areas. **E.** The mean cell area was increased significantly (p<0.0001) in control cells receiving 2 Gy as compared to the unirradiated population. When cells were grown in BuOE there was no significant increase (p=0.99) in cell size following radiation treatment. The combined data of 3 independent experiments is presented here as mean and std dev. Significance was determined using 1-way ANOVA followed by post hoc Tukey's test for multiple comparisons.

Besides increased collagen contraction, activated fibroblasts also display a change in morphology. Specifically, the activated fibroblast becomes larger in size. Thus, we measured cell size of the fibroblasts 48 hours after cells were irradiated with 0 or 2 Gy of radiation and in the presence or absence of MnTnBuOE-2-PyP (Figure 2A, 2B, 2C, 2D, & 2E). We found that irradiating cells with 2 Gy caused a significant increase in mean cell size as compared to control sham unirradiated cells (Figure 2E). However, when cells were irradiated in the presence of MnTnBuOE-2-PyP the size of the fibroblast was not altered (Figure 2E). Thus, MnTnBuOE-2-PyP treatment inhibited the radiation induced increase in cell size.

**Figure 3:**
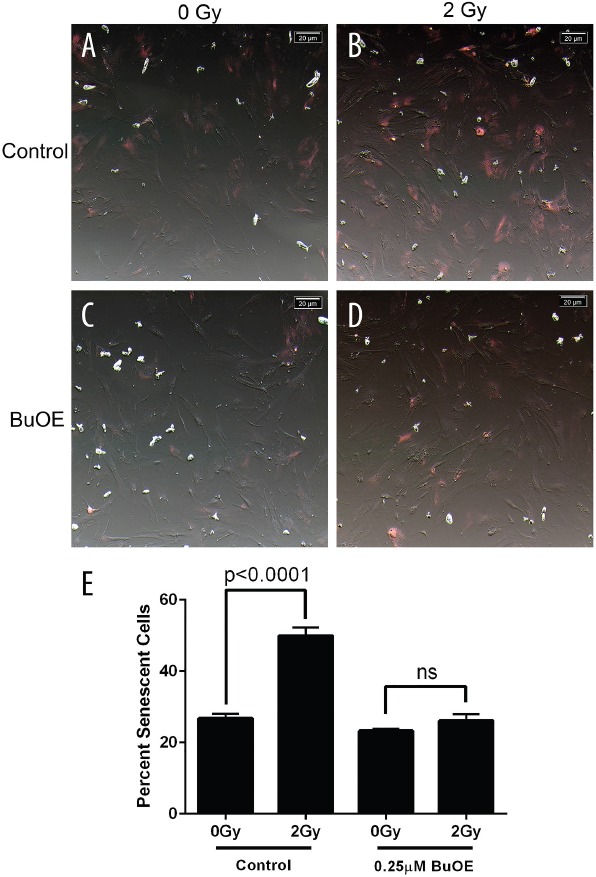
Radiation-induced senescence is reduced in primary colorectal fibroblasts grown in MnTnBuOE-2-PyP Mouse colorectal fibroblasts maintained in media with PBS (Control) or 0.25 μM MnTnBuOE-2-PyP (BuOE) were either sham irradiated **A.** & **C.** or treated with 2 Gy x-rays **B** & **D.**, 48 hours later cells were stained for SA-β-gal expression. Cells were imaged with an inverted brightfield microscope, these images were inverted (A-D) to aid in counting SA-β-gal positive (pink) cells. **E.** Quantification of senescent cells. SA-β-gal expression is significantly increased (p<0.0001) in control cells receiving 2 Gy as compared to the unirradiated population. When cells were grown in BuOE there was no significant increase (p=0.22) in SA-β-gal expression following radiation treatment. All data are representative of the mean and standard deviation and were obtained from 3 independent experiments. The differences of mean percent senescent cells were analyzed for significance using 1-way ANOVA followed by post hoc Tukey's test for multiple comparisons.

Radiation also increases the number of cells undergoing senescence. Two week old fibroblasts were irradiated with 0 or 2 Gy in the presence or absence of MnTnBuOE-2-PyP. Cells were then stained 48 hours after radiation exposure for senescence using a senescence-associated beta-galactosidase (SA-β-Gal) staining solution. Radiation caused over a two fold increase in the percentage of positively stained senescent cells (Figure 3A, 3B, & 3E). However, cells treated with MnTnBuOE-2-PyP were protected from becoming senescent following radiation exposure (Figure 3C, 3D, & 3E). There was no significant difference between the sham irradiated groups and MnTnBuOE-2-PyP irradiated group (Figure 3E). Thus, MnTnBuOE-2-PyP treatment inhibits radiation induced senescence in fibroblast cells as well.

**Figure 4:**
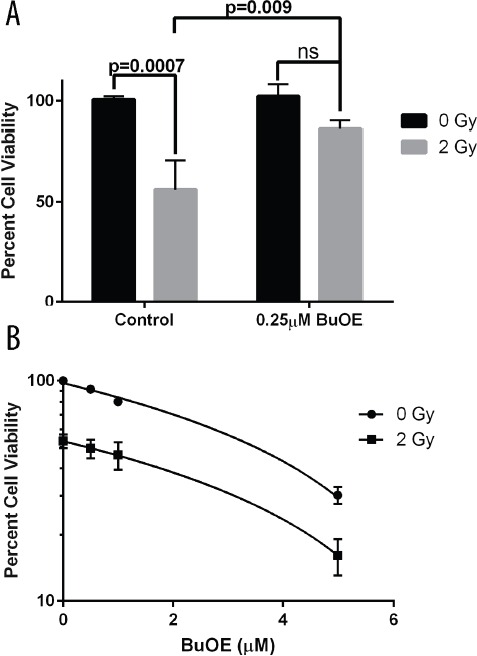
Cell viability following radiation treatment in the presence of MnTnBuOE-2-PyP **A.** Viability of mouse primary colorectal fibroblasts. Fibroblasts were maintained for two weeks in media with PBS (Control) or 0.25 μM MnTnBuOE-2-PyP (BuOE) were either sham irradiated or treated with 2 Gy x-rays. Cultures were assayed for cell viability by measuring the ability to reduce MTT to formazan. Cell viability is significantly decreased (p=0.0007) in control cells receiving 2 Gy as compared to the unirradiated population, but this loss in viability is inhibited with BuOE treatment (p=0.009). Statistical significance and p-values were determined using 1-way ANOVA analysis followed by post hoc Tukey's test for multiple comparisons. **B.** Viability of human colorectal cancer cells. HT-29 cells were either sham irradiated or given 2 Gy x-rays in the presence of increasing doses of BuOE [0, 0.5, 1, 5 μM] Cultures were grown an additional 12 days in the presence of BuOE or PBS for control, then assayed for cell viability measured as the ability to reduce MTT to formazan. BuOE does not protect cancer cells from radiation induced damage (p<0.001) as determined by linear regression analysis of the difference between drug dose response curves with or without radiation treatment. All data are representative of the mean and standard deviation and were obtained from 3 independent experiments.

We also investigated the effects of MnTnBuOE-2-PyP on cell viability. Since a radioprotector should protect normal cells but not tumor cells from radiation damage, we determined the effect of MnTnBuOE-2-PyP on both primary colorectal fibroblasts and colorectal cancer cells (HT-29 cells). We performed an MTT assay to determine the metabolic activity of the cells in the presence or absence of MnTnBuOE-2-PyP and or radiation. We found around a 50% reduction in cell viability (metabolic activity) when fibroblasts were irradiated with 2 Gy (Figure 4A). However, in the presence of MnTnBuOE-2-PyP (0.25 μM) there was no significant decrease in fibroblast viability when these cells were irradiated (Figure 4A). Based on pharmacokinetic data, the highest dose of MnTnBuOE-2-PyP to the colorectal area was 0.5 μM. Therefore, we used this highest dose to determine if there would be any protective effects of MnTnBuOE-2-PyP in combination with radiation on colorectal cancer cells. Radiation (2 Gy) also resulted in a 50% reduction in the viability of colorectal cancer cells (Figure 4B). In contrast to what was observed in normal fibroblasts, MnTnBuOE-2-PyP treated colorectal cancer cells had reduced viability and this effect was further enhanced in combination with radiation. Thus, MnTnBuOE-2-PyP alone or in combination with radiation produces different effects on the viability of normal fibroblasts vs. cancer cells.

**Figure 5:**
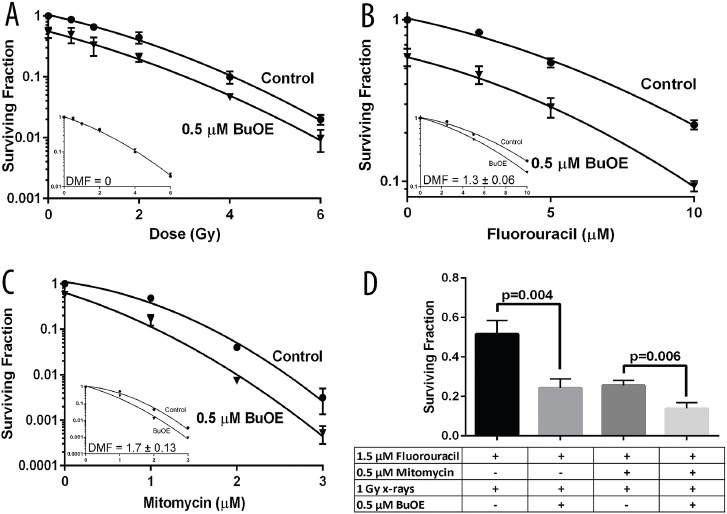
MnTnBuOE-2-PyP does not protect colorectal cancer cells from common therapeutic regimens used clinically for colorectal and anal cancers The effect of MnTnBuOE-2-PyP (BuOE) on dose response to x-radiation or chemotherapy or combinations of those therapies was determined by clonogenic survival. **A.** HT-29 cells growing in log phase were treated overnight with 0.5 μM BuOE then treated with [0, 0.5, 1, 2, 4, 6 Gy] x-rays. The surviving fraction was significantly reduced (p<0.0001) in BuOE treated cells, however the dose response due solely to radiation was not enhanced or diminished by the addition of BuOE (DMF=0, A-inset). **B.** HT-29 cells growing in log phase were treated overnight with 0.5 μM BuOE then treated with [0, 2.5, 5, 10 μM] 5-fluorouracil for 24 hours. The surviving fraction was significantly reduced (p=0.0005) in BuOE treated cells, additionally the dose response due solely to 5-fluorouracil was enhanced by the addition of BuOE (DMF=1.3 ± 0.06, B-inset). **C.** HT-29 cells growing in log phase were treated overnight with 0.5 μM BuOE then treated with [0, 1, 2, 3 μM] Mitomycin for 2 hour. The surviving fraction was significantly reduced (p<0.0001) in BuOE treated cells, the dose response due solely to Mitomycin was enhanced by the addition of BuOE (DMF=1.7 ± 0.13, C-inset). **D.** HT-29 cells were exposed overnight to 0.5 μM BuOE or PBS, cells were then treated with 1.5 μM 5-fluorouracil for 24 hours. Half of those cells were treated with 0.5 μM Mitomycin during the final hour of 5-fluorouracil incubation. All of the samples were irradiated with 1 Gy x-rays and then seeded for clonogenic survival. The combination of 5-fluorouracil and radiation was significantly enhanced in the presence of BuOE (p=0.004) and likewise the combination of 5-fluorouracil, Mitomycin, and radiation was significantly enhanced (p=0.006) by BuOE treatment. All data are representative of the mean and standard deviation and were obtained from 3 independent experiments.

To further demonstrate the anti-tumor effect of MnTnBuOE-2-PyP on colorectal cancer cells, clonogenic assays were performed. In Figure 5A, HT-29 cells grown in the presence of MnTnBuOE-2-PyP produced significantly fewer colonies than HT-29 cells grown without MnTnBuOE-2-PyP. As expected, when the colorectal cancer cells were irradiated with increasing amounts of radiation (0.5 to 6 Gy) there was a dose dependent decrease in colony survival (Figure 5A). When cells were treated with both radiation and MnTnBuOE-2-PyP, the colorectal cancer cells' clonogenicity was further inhibited (Figure 5A). Analyses of the cell survival curves show that normalization to remove the effect of MnTnBuOE-2-PyP alone results in a shared survival curve or a dose modification factor (DMF) of 0 (Figure 5A-inset). Thus, reduced colony formation is an additive effect from MnTnBuOE-2-PyP, and likely not an enhancement of the radiation's ability to reduce clonogenicity. More importantly, this assures that the tumor cells are not being protected from radiation killing.

Because most rectal and anal cancers are treated with radiation and 5-fluorouracil and or Mitomycin C, we wanted to investigate the combined effects of MnTnBuOE-2-PyP and these chemotherapy agents. The addition of 5-fluorouracil (2.5-10 μM) resulted in a dose dependent decrease in cancer cell growth (Figure 5B). In the presence of MnTnBuOE-2-PyP (0.5 μM), the colorectal cancer cell growth was further inhibited. In fact, analyses of the survival curves shows that MnTnBuOE-2-PyP treatment resulted in a DMF of 1.3 ± 0.06, a significant enhancement of the 5-fluorouracil (Figure 5B-inset). A similar dose response was observed for cells treated with 1-3 μM Mitomycin C (Figure 5C). HT-29 cells treated simultaneously with MnTnBuOE-2-PyP (0.5 μM) and Mitomycin C, displayed significantly reduced clonogenicity (DMF = 1.7 ± 0.13) as compared to cells treated with Mitomycin C alone (Figure 5C-inset). Thus, MnTnBuOE-2-PyP in combination with either chemotherapeutic resulted in a reduction of colorectal cancer growth. To determine the effects of radiation combined with chemotherapy and MnTnBuOE-2-PyP, clonogenic assays were performed on cells treated with a combination of chemo-radiation and MnTnBuOE-2-PyP. In order to mimic therapy for rectal cancers, the chemotherapeutic agent, 5-fluorouracil, and radiation were combined with MnTnBuOE-2-PyP and a significant decrease in tumor growth was observed as compared to cell treated with radiation and 5-fluorouracil alone (Figure 5D). When both chemotherapeutics were used in combination with radiation (to mimic anal cancer treatment) and MnTnBuOE-2-PyP treatment, the largest tumor inhibition was observed (Figure 5D). Thus, the addition of MnTnBuOE-2-PyP to standard treatment for rectal or anal cancers does not protect the cancer cells from dying. Instead, there appears to be enhanced inhibition of cancer growth with MnTnBuOE-2-PyP added to the standard therapy.

## DISCUSSION

MnTnBuOE-2-PyP has recently been shown to be an effective radioprotector in head and neck cancer and glioblastoma mouse models [18, 19]. Specifically, MnTnBuOE-2-PyP inhibited inflammation and protected the salivary glands from radiation damage in the head and neck area and MnTnBuOE-2-PyP also protected the white matter from damage in irradiated brains [18, 19]. However, the mechanism by which MnTnBuOE-2-PyP works as a radioprotector has not been determined and needs to be explored so that when these compounds are used clinically we will have a better understanding of their mechanism of action. Although MnTnBuOE-2-PyP is largely understudied, there has been work conducted to understand how other Mn porphyrins protect from radiation damage. The administration of other Mn porphyrins during irradiation of the lung inhibited DNA oxidative damage (8-OhdG) and downregulated TGF-β1, VEGF-A and HIF-1β pathways [33]. It was also shown that these Mn porphyrins inhibit apoptosis in irradiated normal lung tissue by inhibiting PTEN expression [34].

In the present study, we demonstrated that MnTnBuOE-2-PyP inhibited the radiation-induced activation of primary fibroblasts. Specifically, MnTnBuOE-2-PyP inhibited fibroblast morphological changes (increased cell size) in response to radiation and inhibited the ability of the cells to contract their surrounding extracellular matrix. MnTnBuOE-2-PyP also significantly inhibited radiation induced senescence and reduced overall fibroblast cell death caused by radiation exposure. However, MnTnBuOE-2-PyP did not protect the colorectal cancer cells from radiation induced death. In fact, MnTnBuOE-2-PyP appears to produce an additive effect of enhancing radiation induced killing of the colorectal cancer cells. Since rectal and anal cancers are generally treated with radiation and chemotherapy we also investigated the effect of MnTnBuOE-2-PyP in combination with chemo-radiation therapy on colorectal cancer cells. We demonstrated that the addition of MnTnBuOE-2-PyP in combination with both radiation and chemotherapy resulted in further synergistic killing of the colorectal cancer cells.

In order to investigate the possible mechanisms by which MnTnBuOE-2-PyP inhibits radiation induced injury, we developed an *in vitro* system that mimicked the changes seen in radiation fibrosis *in vivo*. We first tried to use immortalized human fibroblasts as a model; however, in our hands these cells did not respond to clinically relevant radiation doses. We found that these immortalized fibroblasts were already in an “activated” state and, thus, did not make for a good model. Therefore, we switched to using primary, mortal, mouse fibroblasts. After isolation from 3-5 colon/rectums, we were able to obtain a total of more than 10 million cells and the cells were viable for 4-5 weeks in culture. At day 5 of culture, we had a pure population of fibroblasts, as determined by immunostaining for fibroblast and epithelial cell markers (data not shown). We found that these primary fibroblasts were viable and did not become activated without stimulus during culture. However, when stimulated with TGF-β1 or radiation, the cells changed morphology and displayed characteristics of an “activated” myofibroblast phenotype. Therefore, we believe that these primary cells represent a good model to test the effectiveness of the radioprotector, MnTnBuOE-2-PyP.

We found that MnTnBuOE-2-PyP significantly inhibited the radiation induced differentiation into either an activated fibroblast or a senescent fibroblast. To our knowledge, this is the first study to demonstrate that manganese porphyrins protect fibroblasts from undergoing activation or senescence. We also noticed when cells were cultured in MnTnBuOE-2-PyP, more fibroblasts were obtained, the cells were viable for a longer period of time, and we observed delayed senescence as compared to fibroblasts cultured without MnTnBuOE-2-PyP (data not shown). It seems that MnTnBuOE-2-PyP inhibits differentiation into other phenotypes and helps to maintain a healthy, normal fibroblast phenotype.

Exactly how MnTnBuOE-2-PyP is inhibiting radiation-induced differentiation into an activated fibroblast or senescent cell remains unclear. MnTnBuOE-2-PyP could be directly scavenging ROS or inhibiting some other stressor signal that promotes differentiation of the fibroblast. Oxidized Mn porphyrins can be readily reduced with small molecule cellular reductants, which spares biological molecules from oxidative damage and allows the Mn porphyrin to further scavenge superoxide [53]. Kobashigawa *et al.* recently published a study showing that the antioxidant, ascorbate, suppresses radiation induced senescence in fibroblasts [9]. They showed that ascorbate inhibited ROS levels immediately after irradiation and that days after irradiation exposure, ascorbate treatment inhibited radiation induced increase in the phosphorylation of p53 and p38 [9]. They postulate that oxidative stress induces p53 accumulation, which results in cellular senescence [9]. Accordingly, in a study investigating the role of another manganese porphyrin, AEOL10150, it was shown that the addition of the porphyrin resulted in a significant decrease in p53 total protein during lung irradiation [34]. Thus, it would be interesting to determine if MnTnBuOE-2-PyP may have similar effects on p53 and this could be one mechanism by which MnTnBuOE-2-PyP may be preventing senescence in colorectal fibroblasts, since p53 induces p21 which is upregulated in senescence.

NADPH oxidase 4 (NOX 4) has also been implicated as a source of ROS in response to irradiation. Park *et al.* demonstrated in lung fibroblasts that ROS generated from NOX4 activates p38 MAPK-Akt signaling pathway, which promotes the differentiation to an activated fibroblast [11]. Therefore, MnTnBuOE-2-PyP could be scavenging the ROS generated by NOX4 and inhibiting the p38 MAPK-Akt pathway and preventing the differentiation into an activated fibroblast. Alternatively, since NF-κB has been shown to activate NOX4 and other Mn porphyrins inhibit NF-κB activity [35], MnTnBuOE-2-PyP could be reducing NOX4 ROS production by direct inhibition of NF-κB.

MnTnBuOE-2-PyP protects the normal tissues from radiation induced damage, but not the tumor. This dual effect of MnTnBuOE-2-PyP has been shown in other studies as well [19]. The role of MnTnBuOE-2-PyP in combination with chemotherapeutic agents has been less studied. To our knowledge, this is the first study to investigate the effects of a manganese porphyrin in combination with Mitomycin C and 5-fluorouracil. We found that and there is a synergistic effect of MnTnBuOE-2-PyP in combination with either Mitomycin C or 5-fluoruracil. When radiation is combined with both chemotherapeutic agents and MnTnBuOE-2-PyP, we found that the synergistic effect is further enhanced.

5-fluorouracil inhibits cancer growth by interfering with RNA synthesis and function, and by inhibiting DNA synthesis [36]. Mitomycin C is an alkylating agent that crosslinks DNA, thereby inhibiting DNA synthesis [37]. However, both of these compounds have also been shown to enhance oxidative stress in cancer cells [38]. There have also been studies to show that certain antioxidants in combination with Mitomycin C or 5-fluorouracil cause further cancer death. Curcumin, in combination with Mytomycin C, was shown to enhance antiproliferative effects on breast cancer cells [39, 40]. The combination of drugs resulted in cell cycle arrest and inhibition of the p38 MAPK pathway [40]. Curcumin and another antioxidant, resveratrol, have been shown to enhance sensitivity of colorectal cancer cells to 5-fluoruracil by inhibiting the NF-κB pathway [41, 42].

Manganese porphyrins have been shown to inhibit HIF-1β and NF-κB, two pathways that tumors exploit to promote survival and progression of the cancer cell [47, 51, 52]. Therefore, one explanation for how MnTnBuOE-2-PyP may be inhibiting cancer growth is through the inhibition of these two pathways. However, recent studies have pointed to another potential mechanism by which manganese porphyrins could be inhibiting tumor growth [28, 29]. Jaramillo *et al.* have shown that the combination of a manganese porphyrin, MnTE-2-PyP, with dexamethasone creates a more oxidizing environment in lymphoma cells [28, 29]. The higher ROS levels created by the combination of MnTE-2-PyP with dexamethasone, results in more apoptosis of the cancer cells [29]. Therefore, in a tumor cell with a higher oxidizing environment, the manganese porphyrin acts as a pro-oxidant rather than an antioxidant, especially when H_2_O_2_ levels are high. Batinic-Haberle *et al.* have postulated that MnTnBuOE-2-PyP and MnTE-2-PyP can undergo oxidation with H_2_O_2_ from Mn^II^P^4+^ to O=Mn^IV^P^4+^ or from Mn^III^P^4+^to O=Mn^V^P=O^3+^ state [30]. The oxidized manganese porphyrins could directly oxidize proteins or lipids if not reduced by cellular reductants, which could result in damage to the cancer cells. More research needs to be conducted in this area to determine exactly how MnTnBuOE-2-PyP is inhibiting colorectal cancer growth in combination with chemo-radiation.

MnTnBuOE-2-PyP has been shown to inhibit head and neck cancer growth in combination with radiation [19] and there have been many papers showing other Mn porphyrins in combination with radiation inhibit tumor growth [28, 29, 43–50]. In the current study, we only show that MnTnBuOE-2-PyP inhibits growth of HT-29 cells. Therefore, we acknowledge that we need to test other colorectal cancer cells in order to show that MnTnBuOE-2-PyP inhibits growth of colorectal cancer.

In summary, this study has demonstrated that MnTnBuOE-2-PyP protects fibroblasts from radiation-induced differentiation. Specifically, MnTnBuOE-2-PyP inhibits both fibroblast activation and senescence due to radiation exposure. MnTnBuOE-2-PyP protects the overall viability of the fibroblast in response to radiation, but does not protect the cancer cells from radiation damage. Furthermore, in combination with MnTnBuOE-2-PyP and chemo-radiation, the colorectal cancers are further sensitized to the therapy. Thus, these preliminary studies indicate that MnTnBuOE-2-PyP helps to protect normal tissues while enhancing the effects of the anti-tumor agents. This dual activity is necessary for a drug to be considered an effective and potent radioprotector.

## MATERIALS AND METHODS

### Experimental animals

8-10 week old male C57BL/6 mice, obtained from Jackson Laboratories, were used in this study. The mice were housed at the University of Nebraska Medical Center (UNMC) and exposed to a 12 h light/12 h dark cycle and fed and watered *ad libitum*. This study was carried out in strict accordance with the recommendations of the Guide for the Care and Use of Laboratory Animals of the National Institutes of Health. All procedures were approved by the institutional animal care and use committee at UNMC (14-054-08-FC).

### Primary colorectal fibroblast isolation

Colons/rectums were isolated from 8-10 week old C57BL/6 mice and digested in 5 mg/mL collagenase I [54]. Cells and large tissue fragments were divided and cultured for 2-3 weeks in Dulbecco's Minimal Essential Media (DMEM) supplemented with 10% fetal bovine serum (FBS), 1% penicillin/streptomyocin and 1% non-essential amino acids with or without 0.25 μM MnTnBuOE-2-PyP. Media and MnTnBuOE-2-PyP were replaced every three days or the cells were passaged at confluency. Cell types in the cultures were characterized using immunofluorescence for Keratin17 (Cell Signaling #4543) to detect contaminating epithelial cells and ERTR7 (Santa Cruz #sc-73355) to verify the presence of fibroblasts. Epithelial cells were not detected in any of the cultures used for experimentation. All experiments were performed in the second or third week of culturing when cell division and viability was maximal. All assays were repeated in triplicate using separate isolations of primary fibroblasts.

### Colorectal cancer cells

HT-29 cells were obtained from the American Type Culture Collection (ATCC # HTB-38). The cells were grown in DMEM media supplemented with 10% FBS, 1% penicillin/streptomyocin and 1% non-essential amino acids. Cells were maintained at 37°C in the presence of 5% CO_2_. Cells were passaged every 2-3 days for no more than 20 passages.

### Collagen contraction assay

Fibroblasts were seeded in T25 flasks at 4 × 10^5^ cells per flask with 5 mL media containing 0.25 μM MnTnBuOE-2-PyP or PBS. Twenty four hours after seeding, the cells were either sham irradiated, given 2 Gy of x-rays (Rad Source RS-2000), or placed in FBS free media with 5 ng/mL of TGFβ1 (BioLegend #580702). Cells were harvested 48 hours after treatment and resuspended at 1 × 10^5^ cells per 50 μL of FBS. Rat tail collagen (Corning #354249) was diluted in 0.5 M NaOH and 10X DMEM to a final concentration of 2 mg/mL. Cells were embedded in the collagen at 1 × 10^5^ cells per 500 μL in a low attachment 24-well plate. Once the collagen discs were solidified, the collagen was released from the sides of the well, and suspended on 500 μL complete growth media. After 12 hours of contraction, the discs were imaged and the area measured using ImageJ software [55].

### Cell size analysis

Fibroblasts were seeded at 2 × 10^5^ cells/flask from cultures grows for two weeks with 0.25 μM MnTnBuOE-2-PyP or an equal volume of PBS for control. The following day, cells were either sham irradiated or treated with 2 Gy x-rays (Rad Source RS-2000). Cells were allowed to grow in 37°C and 5% CO_2_ humidified incubator for 48 hours in the presence of 0.25 μM MnTnBuOE-2-PyP or PBS, then imaged with a Leica inverted phase contract microscope. Images were imported into ImageJ and approximately 1000 cell borders were manually traced per treatment group. Areas were converted to μm^2^ using a conversion factor of 11.8 pixels/μm. Distributions of cell size were analyzed with GraphPad Prism 6 software (La Jolla, California).

### Senescence assay

Fibroblasts were seeded in T25 flasks at 2 × 10^5^ cells per flask with 5 mL media containing 0.25 μM MnTnBuOE-2-PyP or PBS. Twenty four hours after seeding, the cells were either sham irradiated or given 2 Gy of x-rays (Rad Source RS-2000). At 48 hours post-irradiation the cells were washed and fixed in 4% PFA in PBS for 3 minutes. The monolayer was washed twice with PBS for 5 minutes then covered in SA-β-Gal staining solution (0.1% X-gal, 5 mM potassium ferrocyanide, 5 mM potassium ferricyanide, 150 mM sodium chloride, and 2 mM magnesium chloride in 40 mM citric acid/sodium phosphate solution, pH 6.0) [56]. Cells were incubated in a dark, 37°C incubator for approximately 24 hours. Brightfield images of the cell cultures were captured with an Olympus IX-81 inverted microscope. Images were imported into ImageJ, inverted to aid visibility of SA-β-gal positive cells, and enumerated for senescence (pink) staining [55].

### MTT viability assay

Cells were seeded in 96 well plates containing 500 cells/well (HT-29) or 3000 cells/well (fibroblasts). Colorectal cancer cells were treated with 0, 0.5, 1, or 5 μM MnTnBuOE-2-PyP. Fibroblasts were from cultures grown in media containing 0.25 μM MnTnBuOE-2-PyP or PBS for control. Twenty four hours after seeding, the cells were either sham irradiated or given 2 Gy of x-rays (Rad Source RS-2000). Cells were allowed to grow in 37°C and 5% CO_2_ humidified incubator for 12 days in the presence of MnTnBuOE-2-Pyp. The cell monolayer was then washed twice with PBS to remove residual MnTnBuOE-2-PyP and 100 μL of media was added. Next, 55 μL of a 1 mg/mL MTT (3-(4, 5-dimethylthiazolyl-2)-2, 5-diphenyltetrazolium bromide) sterile solution was added to each well [57]. Cells were incubated for 3 hours in a dark 37°C and 5% CO_2_ humidified incubator. Purple formazan crystals were solubilized and the absorbance quantified using a Tecan Infinite200 Pro plate reader. Absorbance at 570 nm with reference at 650 nm were normalized to control and reported as percent viability.

### Clonogenic survival assay

HT-29 cells in log phase of growth were seeded onto 6 well plates and treated overnight with 0.5 μM MnTnBuOE-2-PyP or an equal volume of PBS for control. For the dose response experiments; the following morning, cells were treated with either 5-fluorouracil (APP Pharmaceuticals, Schaumberg, IL) [0, 2.5, 5, 10 μM] for 24 hours, Mitomycin C (Research Products International, Mt Prospect, IL) [0, 1, 2, 3 μM] for 1 hour, or x-rays [0, 0.5, 1, 2, 4, 6 Gy] (Rad Source RS-2000). When all treatments were combined, the cells were treated with 0.5 μM MnTnBuOE-2-PyP overnight, then 1.5μM 5-fluorouracil for 24 hours, 0.5 μM Mitomycin C for the final 1 hour, and lastly 1 Gy x-irradiation. Following treatment, the cells were trypsinized and counted. Serial dilutions were performed and 500 cells were seeded onto well plates in triplicate. Colonies were allowed to form in the presence of 0.5 μM MnTnBuOE-2-PyP for 11 days, then fixed and stained with 0.5% Crystal Violet and 5% methanol. Colonies were observed and counted under a dissecting microscope, only colonies containing >50 cells were counted. The fraction of surviving cells was normalized to plating efficiency of sham treated HT-29 cells.

### Statistical analyses

Statistical analyses were performed using GraphPad Prism 6 Software version 6.0.5 for windows. Reported values are the mean and SEM from three independent experiments, unless otherwise indicated. A one-factor ANOVA followed by a *post hoc* Tukey's test was used to determine significant difference between the mean of multiple comparisons. Dose-response clonogenic cell survival curves were fit to the linear-quadratic model using the non-linear regression tool in Prism. Reported dose modifying factors (DMF) were calculated over the entire cell survival curve from data normalized to remove the effect of MnTnBuOE-2-PyP alone.
